# Ten-Year Follow-Up of Clinical Governance Implementation in Primary Care: Improving Screening, Diagnosis and Control of Cardiovascular Risk Factors

**DOI:** 10.3390/ijerph16214299

**Published:** 2019-11-05

**Authors:** Carina Aguilar Martín, Alessandra Queiroga Gonçalves, Carlos López-Pablo, José Fernández-Sáez, Emma Forcadell Drago, Zojaina Hernández Rojas, Josep Maria Pepió Vilaubí, Dolores Rodríguez Cumplido, Josep Lluis Piñol, Jordi Bladé-Creixenti, Maria Rosa Dalmau Llorca

**Affiliations:** 1 Unitat de Suport a la Recerca Terres de l’Ebre, Fundació Institut Universitari per a la recerca a l’Atenció Primària de Salut Jordi Gol i Gurina (IDIAPJGol), 43500 Tortosa, Tarragona, Spain; caguilar.ebre.ics@gencat.cat (C.A.M.); jfernandez@idiapjgol.info (J.F.-S.); eforcadellg.ebre.ics@gencat.cat (E.F.D.); zojahernandez@gmail.com (Z.H.R.); rdalmau.ebre.ics@gencat.cat (M.R.D.L.); 2 Unitat d’Avaluació, Direcció d’Atenció Primària Terres de l’Ebre, Institut Català de la Salut, 43500 Tortosa, Tarragona, Spain; 3 Unitat Docent de Medicina de Família i Comunitària Tortosa-Terres de L‘Ebre, Institut Català de la Salut, 43500 Tortosa, Tarragona, Spain; 4 Sector de Biologia Molecular i Recerca, Hospital de Tortosa Verge de la Cinta, 43500 Tortosa, Tarragona, Spain; clopezp.ebre.ics@gencat.cat; 5 Equip d’Atenció Primària Tortosa Oest, Institut Català de la Salut, 43500 Tortosa, Tarragona, Spain; jmpepio.ebre.ics@gencat.cat; 6 Equip d’Atenció Primària Tortosa Est, Institut Català de la Salut, 43500 Tortosa, Tarragona, Spain; 7 Fundació Institut Català de Farmacologia, Universitat Autònoma de Barcelona, 08035 Barcelona, Spain; drficf@gmail.com; 8 Equip d’Atenció Primària l’Aldea, Institut Català de la Salut, 43896 Aldea, Tarragona, Spain; jlpinol@semfyc.es; 9 Equip d’Atenció Primària Horts de Miró, Institut Català de la Salut, 43204 Reus, Tarragona, Spain; jblade.tarte.ics@gencat.cat

**Keywords:** cardiovascular diseases, primary health care, clinical audit, feedback, clinical governance, incidence, mortality

## Abstract

Current improvement strategies for the control of cardiovascular risk factors (CRFs) in Europe are based on quality management policies. With the aim of understanding the effect of interventions delivered by primary healthcare systems, we evaluated the impact of clinical governance on cardiovascular health after ten years of implementation in Catalonia. A cohort study that included 1878 patients was conducted in 19 primary care centres (PCCs). Audits that comprised 13 cardiovascular health indicators were performed and general practitioners received periodic (annual, biannual or monthly) feedback about their clinical practice. We evaluated improvement in screening, diagnosis and control of the main CRFs and the effects of the feedback on cardiovascular risk (CR), incidence of cardiovascular disease (CVD) and mortality, comparing baseline data with data at the end of the study (after a 10-year follow-up). The impact of the intervention was assessed globally and with respect to feedback frequency. General improvement was observed in screening, percentage of diagnoses and control of CRFs. At the end of the study, few clinically significant differences in CRFs were observed between groups. However, the reduction in CR was greater in the group receiving high frequency feedback, specifically in relation to smoking and control of diabetes and cholesterol (Low Density Lipoprotein (LDL) and High Density Lipoprotein (HDL)). A protective effect of having a cardiovascular event (hazard ratio (HR) = 0.64, 95% confidence interval (CI) = 0.44–0.94) or death (HR = 0.55, 95% CI = 0.35–0.88) was observed in patients from centres where general practitioners received high frequency feedback. Additionally, these PCCs presented improved cardiovascular health indicators and lower incidence and mortality by CVD, illustrating the impact of this intervention.

## 1. Introduction

Cardiovascular disease (CVD) causes one in three deaths worldwide [1]. In Europe, 45% of deaths are attributable to CVD [2], and in Spain, the first specific cause of death is ischaemic heart disease (14.6%) [3]. The most recent European and American clinical guidelines for CVD prevention recommend the control of the main modifiable cardiovascular risk factors (CRFs) for the prevention of CVD, namely smoking, arterial hypertension (AHT), diabetes mellitus (DM), dyslipidaemia, obesity, unhealthy eating habits and physical inactivity [4,5]. The estimated prevalence of CRFs among Spanish individuals between 35–74 years of age ranges from 11% to 47% [6]. Developing strategies to improve control of CRFs in primary care remains essential, since in Spain, prevention and management of CVD occurs mainly in primary care centres (PCCs) [7].

Current improvement strategies for the control of CRFs in Europe are based on quality management policies [8]. Clinical governance emerged in the United Kingdom in 1998 and was defined as a framework for the organisations of the National Health Service to continuously improve the quality of its services and to guarantee high standards of care [9]. Clinical governance has since expanded to other settings, and some factors have been identified as crucial to its effectiveness, especially audits, evidence-based practice, risk management, monitoring the outcomes of medical care, continuing medical education and systems for managing poor performance [10,11,12,13,14,15,16,17,18].

Audits and feedback are widely used strategies for improving clinical practice [19,20]. In an audit and feedback process, the professional practice or productivity is measured and compared with professional standards or specific targets that need to be met. This type of intervention is based on the assumption that health professionals are motivated when they receive feedback about their practice [20].

Previous studies have concluded that the effects of audit and feedback are generally small to moderate [20,21,22]. To date, it remains unclear how effective audits and feedback are at improving clinical practice, and which characteristics of audits and feedback have the greatest impact [23,24]. Several studies conducted in the primary care setting have attempted to determine the effects of audit and feedback interventions in cardiovascular health [25,26,27,28,29,30,31]. In an article that evaluated the effects of audit and feedback on secondary prevention in patients with non-acute stroke, blood pressure and metabolic control were considered adequate in 73% of patients with hypertension and 62% of patients with diabetes, respectively [25]. In patients from Western Cape (South Africa) with diabetes, implementing audit and feedback systems improved the result of seven out of nine clinical indicators [26]. Hickey et al. [28] reported a significant improvement in 17 of 40 clinical indicators of patients with acute coronary syndrome and congestive heart failure. In contrast, two interventions for the management of hypertension in primary care reported scarce [29] or no improvement [30] in the control of blood pressure using audit and feedback strategies.

The Catalan Institute of Health (CIH) implemented clinical governance in primary care in 2001 for the continuous improvement of its services and the translation of scientific evidence to clinical practice [32]. The current study aimed to evaluate the impact of clinical governance on cardiovascular health in the PCCs of the CIH in Tarragona (Catalunya, Spain) 10 years after its implementation, focusing on the post-audit feedback offered to general practitioners about their clinical practice. To this end, improvements in screening, diagnosis and control of the main CRFs and the effects of feedback on cardiovascular risk (CR), CVD incidence and mortality were evaluated. Specifically, we evaluated some modifiable CRFs, endorsed by scientific evidence [4,5], that are considered quality indicators in clinical governance, and which might have a positive impact on cardiovascular health when used in feedback reports.

## 2. Material and Methods

### 2.1. Study Design and Participants

A cohort study was conducted in a population between 35 and 75 years of age from the 19 CIH primary care centres (PCC) of Tarragona, Spain, in 2001 (patients with at least one visit during this year were included).

### 2.2. Sample Size and Patient Selection

To estimate the sample size of the feedback study, the parameters used for the detection of a CVD rate ratio of 0.85 (at 10 years of tracking) between the two groups were: an alpha risk of 0.05, a beta risk of 0.20 and a ratio of 1:1 between the two groups, assuming a rate of incidence of CVD in the general population of 0.085. The number of patients calculated with the Fleiss correction was at least 1550 (775 per group). A conglomerate sampling was conducted considering each PCC as a cluster. In each PCC, a randomised selection of patients was performed, in order to achieve a similar number of patients by PCC. The final number of participants was 1878.

### 2.3. Data Collection

Audits have been performed by the Information System Unit of the CIH Tarragona-Terres de l’Ebre since 2001, when clinical governance was implemented. The information generated by the audits was sent to PCC managers, which then gave the feedback to the health professionals (general practitioners and nurses). Thirteen indicators from the clinical governance contract related to cardiovascular health were used for the feedback (screening for CVD, CVD risk factors and control of CRFs). The feedback varied depending on the frequency of delivery by the PCC managers (annually, biannually or monthly), and on whether the medical records of the PCC were computerized.

Data were collected at baseline (2001) and at the end of the study (10 years after implementing clinical governance) by trained professionals from each PCC. All information related to patients and health indicators were collected from primary healthcare clinical records.

### 2.4. Variables

Intensity of feedback: this variable was constructed based on feedback data from the first three years of follow-up (2001 a 2004) and was categorized as: a) High Frequency: when the indicators of the clinical governance contract were provided either monthly or biannually to health professionals by the PCC manager; and b) Low Frequency: when the indicators of the clinical governance contract were provided annually to health professionals by the PCC manager.

Outcome variables: incidence of CVD (acute myocardial infarction, angina pectoris, stroke or peripheral artery disease); incidence of mortality; diagnosis of CRFs (AHT, DM, dyslipidaemia and smoking); screening for CRFs (blood pressure, blood glucose, total cholesterol, Low Density Lipoprotein (LDL) cholesterol, High Density Lipoprotein (HDL) cholesterol and smoking, measured at least the previous two years); control of CRFs (glycated haemoglobin (HbA1c) < 7%; blood pressure less than 140/90 mmHg or less than 130/85 mmHg if diagnosed with DM or CVD; total cholesterol less than 200 mg/dL, LDL cholesterol below 130 mg/dL; non-smoker); and CR according to the Framingham Risk Score (high CR was considered as having a score equal or greater than 20).

The secondary variables analysed were: sociodemographic characteristics of the patients (age, gender); rural or urban PCC; and medical residents (MRs) teaching centre.

### 2.5. Statistical Analysis

The impact of implementing clinical governance was statistically evaluated in the whole study population firstly globally, and secondly with respect to the feedback frequency (classified as high or low) received by the 96 general practitioners participating in the study.

The global impact of implementing clinical governance at the end of follow-up on screening, diagnosis and control of CRFs and CR was evaluated with McNemar’s test for qualitative variables, and with Student’s *t*-test for paired samples for quantitative variables. Conversely, to compare these variables with respect to feedback frequency, we used Pearson chi-squared test for qualitative variables, and Student’s *t*-test for independent samples for quantitative variables.

The rates of CVD incidence in the groups with high (I1) and low (I0) feedback frequency and their rate ratio (RRt = I1/I0) were calculated. A Cox regression model was used to identify the factors determining the incidence of CVD and mortality at 10 years of follow-up. Significance level was set at 5%. IBM SPSS Statistics v.23.0 (IBM, Armonk, NY, USA) for Windows was used for statistical analysis.

### 2.6. Ethics

This study was approved by the Clinical Research Ethics Committee of the Fundació Institut Universitari per a la Recerca a l’Atenció Primària de Salut Jordi Gol i Gurina (P16/099).

## 3. Results

Of the 1878 patients selected at the beginning of the study, a final sample of 1719 patients (706 with low feedback frequency and 1013 with high feedback frequency) was achieved in 2011. Losses were due to 94 deaths and to 65 participants lost to follow-up. With respect to the 19 participating PCCs, 11 received high feedback frequency, nine of which were rural PCCs, three provided MR training, and four were computerised at the beginning of the study. Table 1 shows the general characteristics of the 19 PCCs.

The overall effect of implementing clinical governance is shown in Table 2, which summarises the variables and CRFs evaluated in patients at the beginning and end of the study. After 10 years, CR, screening, control and diagnosis of all CRFs improved significantly. Notably, screening for smoking increased from 9.5% in 2001 to 91.2% in 2011.

Taking into account the characteristics of the PCCs at baseline, the two cohort groups (high and low feedback) showed no statistically significant differences in the percentage of rural centres, provision of MR training, computerisation, mean number of general practitioners and assigned population ≥14 years old by general practitioner. The only significant difference was the assigned population ≥65 years old by general practitioner, which was higher in the group with a low frequency of feedback (*p* = 0.044).

Table 3 shows the results of the patients with respect to feedback frequency. When comparing the age of participants in relation to feedback frequency, it is clear that the mean age is significantly higher in the low feedback frequency at the beginning of the study and throughout the 10-year follow up. The percentage of women was not significantly different at the beginning but approached significance at the end of the study. The initial differences in arterial hypertension disappear over the 10-year follow-up, and the significant difference in diastolic blood pressure observed at the end of the study is not considered clinically relevant (75.3 versus 76.7). Compared with the high frequency feedback group, the percentage of patients with diabetes was significantly higher in the low frequency feedback group at the end of follow-up (*p* = 0.018). With respect to dyslipidaemia, the only statistically significant differences found at the end of follow-up were in total cholesterol (*p* = 0.007) and HDL (*p* < 0.001), although these differences were not considered clinically relevant. In the beginning, the percentage of smokers was higher in the high frequency feedback group, and a greater than two-fold reduction in smoking was achieved in this group (27.0 versus 19.4) compared to the low frequency feedback group (15.4 versus 13.1). Finally, there was evidence of a greater reduction of CR (*p* = 0.001) and a lower incidence of CVD (*p* = 0.011) and mortality in the high frequency feedback group (*p* < 0.001).

The possible association of the variables with CVD and mortality was detected by a Cox regression (Table 4 and Table 5). The factors associated with greater risk of CVD in the final multivariate model were older age (hazard ratio (HR) = 1.05, 95% confidence interval (CI) = 1.03–1.07), being male (HR = 1.98, 95% CI = 1.39–2.82), having a diagnosis of AHT (HR = 1.74, 95% CI = 1.21–2.51) or DM (HR = 1.87, 95% CI = 1.28–2.75) at the beginning of the study and being assigned to a doctor who does not teach MR (HR = 1.55, 95% CI = 1.06–2.28). The only protective variable found was to belong to the group of patients whose health professionals and centres received high frequency feedback (HR = 0.64, 95% CI = 0.44–0.94) (Table 4). With respect to mortality, the factors associated with a greater risk of death included older age (HR = 1.13, 95% CI = 1.10–1.17), rural location (HR = 2.03, 95% CI = 1.27–3.23), having a diagnosis of DM (HR = 1.75, 95% CI= 1.14–2.71) and being a smoker at the beginning of the study (HR = 2.01, 95% CI = 1.19–3.40). As with CVD, the only protective variable was belonging to the group of patients whose professionals and centres received high frequency feedback (HR = 0.55, 95% CI= 0.35–0.88) (Table 5).

## 4. Discussion

Audit and feedback are considered interventions founded on the principle of collective responsibility [20]. The hypothesis proposed in the current study is based on the assumption that professionals receiving more feedback will improve their medical practice, with direct, positive repercussion on the health of their patients.

Audit and feedback are described as interventions that lead to small but potentially important improvements in professional practice [33]. In this study, we evaluated the effect of implementing clinical governance within the context of cardiovascular health and observed the benefits of audits and feedback on the most important modifiable CRFs. Ten years after the implementation of the model, a general improvement was found with respect to screening, percentage of diagnoses and control of CRFs. When evaluating the CR of the population as measured by the Framingham Risk Score, we observed a reduction in risk, which we attribute to the improved control of CRFs during the same period.

Studies conducted in the primary care setting have reported positive effects from audits and feedback, with improvements in the indicators of cardiovascular control in patients with acute coronary syndrome [28], diabetes [26] and AHT [34]. In contrast, other studies have not found clear benefits of this strategy for the control of blood pressure in hypertensive patients, or for the prevention of CVD [29,30].

In our study, few clinically relevant differences in CRFs were found between the groups compared (low or high feedback) at the end of the study. However, the reduction in CR was greater in the group receiving high frequency feedback. Additionally, although the compliance with the quality standard was low at the beginning in both groups, a greater improvement was observed in the group receiving high frequency feedback, specifically with regard to smoking, control of diabetes and of LDL and HDL cholesterol. This effect had also been reported in previous studies, which concluded that both the absolute effect and the relative effectiveness of audit and feedback are greater when compliance of the recommended practice is low at the beginning and the feedback is more intense [21,22]. Other studies have reported the beneficial effects of audit and feedback on smoking, lipid control and metabolic control of diabetes in patients with acute coronary syndrome, ictus and diabetes [25,26,28].

One factor that might have contributed to the improvement observed in the quality indicators is the computerisation of PCCs. In this study, the improvement of health results was greater in PCCs with high frequency of feedback. We believe that part of this effect is due to the larger frequency of computerisation in these PCCs at the beginning of the study. We believe that computerisation exposed the PCCs to a greater intervention intensity and could, therefore, directly influence improvements in the management of the PCCs, in clinical practice and consequently in the health results. This effect was also observed in another study in which computerisation enabled better audits and feedback of different quality indicators [35].

One limitation of this study is that the feedback intensity was probably not constant throughout the years of implementing clinical governance, since the computerisation of the centres was gradual [36]. However, both study groups showed improvements in most indicators at the end of the study, which we attribute to the intervention.

The benefit of CR reduction is apparent in the incidence rate of CVD calculated in the 10-year period, which was lower than that calculated in Spain a year after the study began. In 2002, the incidence rate of CVD in Spain was 14.1 CVD/1000 inhabitants [37], compared to 9.6 CVD/1000 in our study, which represents a reduction of almost five new cases per 1000 inhabitants. This reduction should represent an improvement in the quality of life of patients and economic savings of medical and pharmaceutical resources.

With regard to factors associated with risk of CVD after adjusting by other variables, we found that high frequency feedback had an independent and statistically significant protective effect. Another protective variable was belonging to a PCC with specialised training of MR. Some risk factors identified for CVD were: being male, older age, and a diagnosis of AHT and diabetes.

High frequency feedback also had a protective effect on mortality. We hypothesise that this reduced risk can be attributed to the better control of CRFs. Regarding risk factors, smoking and diabetes were identified as risk factors for mortality. It has been observed that the reduction of smoking and diabetes correlates with the constant decline of the mortality rate by CVD worldwide [38]. A study by Unal et al. observed that modest reductions in CRFs resulted in a gain of up to four years of life [39].

Another limitation of this study are potential confounding factors related to the characteristics of the PCCs that may affect the results and that were not taken into account in the study, for instance doctor-to-patient ratio, difference in resources, post-graduate qualifications of the general practitioners and staff turnover rate. Future studies should take these factors into account.

This study started when the new information and communications technologies were gradually incorporated in the primary care consultation rooms of PCCs from Catalonia. Future comparative studies on clinical governance should consider the changes that occur over time. In the case of this study, we experienced the computerisation of clinical records and the use of platforms linked to the electronic clinical records that follow up clinical indicators (and that facilitate constant feedback to clinicians), continuing training of professionals and changes in health policies.

## 5. Conclusions

The implementation of clinical governance in CIH primary care centres in the province of Tarragona improved the screening and control of CRFs. In addition, we observed that the group receiving high feedback frequency showed a greater reduction in CR and a lower incidence of CVD and mortality. These results demonstrate the benefits of this strategy on the cardiovascular health of the population studied. Importantly, primary healthcare professionals should receive feedback on indicators related to prevention of hypertension, diabetes and smoking. Continuing training of professionals should be stressed, since teaching centres obtained better results. Further studies should evaluate the efficiency of continuous monitoring and feedback of clinical practice indicators in primary care, and which indicators correlate best with a positive, long-term impact on the health of patients.

## Figures and Tables

**Table 1:** General description of the characteristics of the 19 healthcare centres participating in the study and the number of patients analysed in each centre.

Centre	Feedback Intensity	Setting	Number of GPs ^a^	MR ^b^ Teaching	Computerization	Population ≥14 years	Population ≥65 years	Number of Patients
1	Low	Rural	4	No	No	7163	1018	98
2	Low	Urban	14	No	Yes	23,009	2877	100
3	Low	Rural	5	Yes	No	9208	1211	101
4	Low	Rural	12	No	No	10,923	1823	97
5	Low	Urban	17	No	No	22,861	3168	102
6	Low	Rural	6	No	No	10,131	1071	101
7	Low	Rural	10	No	No	9946	1344	100
8	Low	Rural	7	No	No	7404	1211	100
9	High	Rural	4	No	No	6589	652	102
10	High	Urban	10	No	No	17,270	1885	100
11	High	Urban	10	Yes	No	15,169	1540	99
12	High	Urban	9	No	Yes	12,205	919	101
13	High	Urban	7	No	No	10,948	996	99
14	High	Urban	6	No	No	10,515	699	100
15	High	Rural	3	No	No	4297	463	101
16	High	Rural	5	No	Yes	5631	678	100
17	High	Urban	11	Yes	No	20,709	1592	100
18	High	Urban	4	No	No	5400	356	100
19	High	Urban	10	No	Yes	15,265	1569	77

^a^ General practitioners; ^b^ Medical resident.

**Table 2:** Sociodemographic characteristics and indicators of health, cardiovascular disease and mortality at baseline and at of the 10-year follow-up.

Variable	2001	2011	*p* Value
	(*N* = 1878)	(*N* = 1719)	
Age (ẋ ± ŝ)	54.7 ± 11.3	64.7 ± 11.3	<0.001 ^a^
Gender (*n* (%) of women)	1026 (54.6)	956 (55.6)	0.591 ^b^
AHT screening (*n* (%) of yes)	1381 (73.5)	1468 (85.4)	0.001 ^b^
Diagnosis of AHT (*n* (%))	578 (30.8)	860 (49.9)	<0.001 ^b^
Control of AHT (*n* (%))	749 (39.9)	978 (56.9)	<0.001 ^b^
Diabetes screening (*n* (%)of yes)	1223 (65.1)	1430 (76.1)	<0.001 ^b^
Diagnosis of diabetes (*n* (%))	267 (14.2)	391 (22.8)	<0.001 ^b^
Control of diabetes (*n* (%))	87 (50.0)	134 (63.5)	0.010 ^b^
Dyslipidaemia screening (*n* (%) of yes)	1250 (66.6)	1432 (76.3)	<0.001 ^b^
Diagnosis of dyslipidaemia(*n* (%))	578 (30.8)	931 (54.2)	<0.001 ^b^
Control of total cholesterol (*n* (%))	936 (74.5)	1288 (89.9)	<0.001 ^b^
Control of LDL cholesterol (*n* (%))	457 (72.3)	1131 (86.3)	<0.001 ^b^
Screening for smoking (*n* (%) of yes)	179 (9.5)	1567 (91.2)	<0.001 ^b^
Smoking (*n* (%))	415 (22.1)	272 (16.7)	<0.001
Percentage cardiovascular risk (ẋ ± ŝ)	10.4 ± 7.9	8.8 ± 7.1	<0.001 ^a^
High cardiovascular risk (*n* (%))	233 (13.1)	135 (8.3)	0.004 ^b^
Diagnosis of CVD (*n* (%))	109 (5.8)	260 (15)	<0.001 ^b^
Cumulative incidence of CVD (*n* (%)]		163 (9.2)	
CVD rate per 1000 person-years (95% CI)		9.6 (8.2–11.3)	
Cumulative incidence of mortality (*n* (%) of yes)		94 (5.2)	
Mortality rate per 1000 person-years (95% CI)		8.6 (7.3–10.1)	

ẋ = mean; ŝ = standard deviation; *n* = number of individuals or cases; % = percentage; AHT = arterial hypertension CVD = cardiovascular disease; LDL: low density lipoprotein; CI: confidence interval. ^a^ Student’s *t*-test for paired samples; ^b^ McNemar test.

**Table 3:** Sociodemographic characteristics and indicators of health, cardiovascular disease and mortality at baseline and at of the 10-year follow-up according to feedback intensity.

Variable	2001			2011		
	Feedback Intensity			Feedback Intensity		
	Low	High	*p* Value	Low	High	*p* Value
	(*N* = 798)	(*N* = 1080)		(*N* = 706)	(*N* = 1013)	
Age (ẋ ± ŝ)	56.8 ± 11.2	53.4 ± 11.2	<0.001 ^a^	66.8 ± 11.2	63.4 ± 11.2	<0.001 ^a^
Gender (*n* (%) of women)	424 (53.1)	602 (55.7)	0.262 ^b^	385 (54.5)	571 (56.3)	0.048 ^b^
Percentage cardiovascular risk (ẋ ± ŝ)	10.8 ± 8.0	10.1 ± 7.8	0.052 ^a^	10.3 ± 7.6	7.8 ± 6.4	<0.001 ^a^
Reduction in cardiovascular risk (ẋ ± ŝ)				−0.15 ± 4.4	1.77 ± 5.4	0.001 ^a^
Diagnosis of AHT (*n*(%))	269 (33.7)	309 (28.6)	0.018 ^b^	364 (49.9)	496 (49.9)	0.514 ^b^
Systolic blood pressure mmHg (ẋ ± ŝ)	135.4 ± 18.8	131.6 ± 17.9	<0.001 ^a^	132.5 ± 14.9	131.9 ± 16.5	0.506 ^a^
Diastolic blood pressure mmHg: (ẋ ± ŝ)	80.6 ± 10.4	79.8 ± 10.4	0.147 ^a^	75.3 ± 10.0	76.7 ± 10.6	0.009 ^a^
Control blood pressure (*n* (%))	262 (48.8)	487 (57.8)	0.001 ^b^	387 (69.1)	591 (65.1)	0.113 ^b^
Diagnosis of diabetes (*n* (%))	122 (15.3)	145 (13.4)	0.253 ^b^	186 (25.6)	205 (20.7)	0.018 ^b^
HbA1c in diabetics (ẋ ± ŝ)	6.9 ± 2.03	7.4 ± 1.8	0.088 ^a^	6.7 ± 1.3	6.5 ± 1.7	0.524 ^a^
Control of diabetes (*n* (%))	48 (55.2)	39 (44.8)	0.172 ^b^	93 (60.4)	41 (71.9)	0.122 ^b^
Diagnosis of dyslipidaemia (*n* (%))	233 (29.2)	345 (31.9)	0.202 ^b^	403 (55.5)	528 (53.2)	0.337 ^b^
Total cholesterol mg (ẋ ± ŝ)	223 ± 45	225 ± 40	0.365 ^a^	196.3 ± 39.1	202.1 ± 40.1	0.007 ^a^
Control total cholesterol (*n* (%))	410 (76.9)	526 (72.7)	0.086 ^b^	493 (90.0)	795 (89.9)	0.985 ^b^
HDL cholesterol mg (ẋ ± ŝ)	60 ± 16	55 ± 14	<0.001 ^a^	49.1 ± 15.0	53.4 ± 14.2	<0.001 ^a^
LDL cholesterol mg (ẋ ± ŝ)	133 ± 38	141 ± 35	<0.012 ^a^	124.2 ± 34.6	123.1 ± 33.3	0.573 ^a^
Control LDL cholesterol (*n* (%))	243 (75.7)	214 (68.8)	0.053 ^b^	439 (84.3)	692 (87.7)	0.076 ^b^
Smoking (*n* (%))	123 (15.4)	292 (27.0)	<0.001 ^b^	88 (13.1)	184 (19.4)	<0.001 ^b^
Diagnosis of CVD (*n* (%))	45 (5.6)	64 (5.9)	0.798 ^b^	126 (17.5)	134 (13.2)	0.017 ^b^
Cumulative incidence of CVD (*n* (%))				83 (12.2)	80 (8.4)	0.011 ^b^
CVD rate per 1000 person-years (95% CI)				11.7 (9.3–14.5)	8.2 (6.5–10.3)	
Ratio of CVD rates (95% CI)				0.706 (0.519–0.960)		0.025
Cumulative incidence of mortality (*n* (%) of yes)				65 (8.4)	29 (2.8)	<0.001 ^b^
Mortality rate per 1000 person-years (95% CI)				8.4 (6.5–10.8)	2.7 (1.8–3.9)	
Mortality rates (95% CI)				0.326 (0.210–0.505)		

ẋ = mean; ŝ = standard deviation; n = number of individuals or cases; % = percentage; AHT = arterial hypertension; CVD = cardiovascular disease; HbA1c = glycated haemoglobin; LDL = low density lipoprotein; HDL = high density lipoprotein; CI = confidence interval. ^a^ Student’s *t*-test for independent samples; ^b^ Pearson chi-square test.

**Table 4:** Association between cardiovascular disease and sociodemographic characteristics and indicators of health by multiple Cox regression.

Factors	Univariate Model			Multivariate Model		
Variable (Reference Group)	HR	95% CI	*p* Value	HR	95% CI	*p* Value
Age	1.07	1.05–1.08	<0.001	1.05	1.03–1.07	<0.001
Gender (Female)	2.28	1.66–3.15	<0.001	1.98	1.39–2.82	<0.001
High cardiovascular risk (CR ≤ 20)	3.87	2.78–5.39	<0.001			
Location (Urban)	1.49	1.09–2.03	0.013			
Diagnosis of AHT (No diagnosis)	2.67	1.96–3.64	<0.001	1.74	1.21–2.51	0.003
Diagnosis of DM (No diagnosis)	3.09	2.19–4.35	<0.001	1.87	1.28–2.75	0.001
Diagnosis of dyslipidaemia (No diagnosis)	1.81	1.32–2.47	<0.001			
Feedback frequency (Low)	0.64	0.47–0.88	0.005	0.64	0.44–0.94	0.024
Cardiovascular risk	1.08	1.07–1.10	<0.001			
Teaching of MR (Yes)	1.09	0.77–1.54	0.623	1.55	1.06–2.28	0.025

CR: cardiovascular risk; AHT: arterial hypertension; DM: diabetes mellitus; MR: medical resident; HR: Hazard ratio; CI: confidence interval.

**Table 5:** Association between mortality and sociodemographic characteristics and indicators of health by multiple Cox regression.

Factors (Reference Group)	Bivariate Model			Multivariate Model		
	HR	95% CI	*p* Value	HR	95% CI	*p* Value
Age	1.14	1.10–1.17	<0.001	1.13	1.10–1.17	<0.001
Gender (Female)	1.33	0.87–2.04	0.194			
High cardiovascular risk (CR ≤ 20)	3.61	2.27–5.75	<0.001			
Location (Urban)	2.60	1.63–4.15	<0.001	2.03	1.27–3.23	0.003
Diagnosis of AHT (No diagnosis)	2.95	1.92–4.52	<0.001			
Diagnosis of DM (No diagnosis)	3.02	2.01–5.12	<0.001	1.75	1.14–2.71	0.011
Diagnosis of dyslipidaemia (No diagnosis)	1.11	0.70–1.77	0.646			
Feedback frequency (Low)	0.35	0.22–0.55	<0.001	0.55	0.35–0.88	0.012
Cardiovascular risk	1.08	1.05–1.10	<0.001			
Smoking (Non-smoker)	1.10	0.65–1.88	0.713	2.01	1.19–3.40	0.009

CR: cardiovascular risk; AHT: arterial hypertension; DM: diabetes mellitus; HR: Hazard ratio; CI: confidence interval.
